# Atorvastatin Improves the Propionic Acid-Induced Autism in Rats: The Roles of Sphingosine-1-Phosphate and Anti-inflammatory Action

**DOI:** 10.7759/cureus.36870

**Published:** 2023-03-29

**Authors:** Ferit Durankuş, Korkut Budak, Yakup Albayrak, İbrahim H Sever, Bahattin Özkul, Yigit Uyanıkgil, Neslihan Albayrak, Oytun Erbas

**Affiliations:** 1 Medical Education, Medeniyet University, İstanbul, TUR; 2 Medicine, Tekirdağ Namık Kemal University, Tekirdağ, TUR; 3 Medical Education, Tekirdağ Namık Kemal University, Tekirdağ, TUR; 4 Medicine, Demiroğlu Bilim University, İstanbul, TUR; 5 Medicine, İstanbul Atlas University, İstanbul, TUR; 6 Histology and Embryology, Ege University Faculty of Medicine, Izmir, TUR; 7 Medicine, Çorlu State Hospital, Tekirdağ, TUR; 8 Department of Physiology, Istanbul Bilim University School of Medicine, Istanbul, TUR

**Keywords:** hippocampus, lactate, sphingosine, atorvastatin, autism

## Abstract

Purpose

The aim of this study is to investigate the benefits of atorvastatin on the propionic acid-induced autism model via increasing sphingosine-1-phosphate and anti-inflammatory actions with imaging and brain tissue investigations.

Materials and methods

Twenty-five mg/kg/day/rat of propionic acid (PPA) was administered intraperitoneally to 20 male Wistar rats, and 10 male Wistar rats were fed orally. Study groups were designed as follows: Group 1: Control Group (orally fed control, n=10); Group 2 (PPA+saline, n=10); Group 3 (PPA+Atorvastatin, n=10). The brain biochemical and histopathology assessments and magnetic resonance (MR) imaging were conducted across groups in order to compare them.

Results

The PPA+Atorvastatin group was found to have significantly lower levels of brain malondialdehyde, IL-2 level, IL-17, tumor necrosis factor-alpha (TNF-α), and lactate compared to the PPA+saline group. The PPA+Atorvastatin group had higher levels of nerve growth factor and nuclear factor erythroid 2-related factor 2 (NRF-2) and sphingosine-1-phosphate. In histopathology assessments, the PPA+Atorvastatin group was found to have significantly higher neuronal counts of CA1 and CA2 in the hippocampus, and Purkinje cells in the cerebellum.

Conclusions

Current findings suggest that atorvastatin increases sphingosine-1-phosphate levels and decreases inflammatory actions which characterize the autism rodent model implemented in this study. These preliminary results have to be confirmed by further experimental and clinical studies.

## Introduction

Autism Spectrum Disorder (ASD) is distinguished by impaired social behavior and interaction, and difficulties with interests and activities. Developmental language and motor abnormalities are typical clinical signs in individuals with ASD. The estimated rate of ASD is reported as 1 in 150 children globally. Males tend to be affected with ASD nearly three times more than females and the prevalence varies by ethnicity [1].

The exact etiology of ASD is unclear. Moreover, the current treatments are far from healing the core symptoms regarding the limited knowledge of etiology [2]. The animal models for ASD are considered to be important for the development of new treatment strategies, monitoring the effects and side effects of new agents, and understanding the exact pathophysiology. Animal models of ASD implemented by administering propionic acid (PPA) have been proposed to generate ASD symptoms and neuro-inflammatory changes in the rat model of ASD [3]. PPA is a product of enteric bacteria and it is able to reach the brain by crossing the gut-blood and gut-brain barriers. PPA affects the neurons by accumulation in the cells; thus, it can affect the functional process of a number of neurotransmitters [4]. A larger amount of PPA (e.g. 4 μl of 0.26 M solution) is reported to be positively associated with the development of systematic mitochondrial dysfunction by increasing the level of free acyl-carnitine in experimental studies. Moreover, it was reported that thirty percent of ASD patients had elevated levels of carnitine-bound unprocessed long-chain and very-long-chain fatty acids which provided the possible association between PPA and ASD [5].

Statins, 3-hydroxy-3-methyl-glutaryl-CoA (HMG-CoA) reductase inhibitors, are commonly used for the management of cholesterol-lowering states and the primary aim of the statins is decreasing mortality and morbidity which are sourced from atherosclerosis and cardiovascular events. The possible mechanisms of the neuroprotective effects of statins are considered to be associated with reducing the amount of isoprenoids and increasing nitric oxide synthase which leads to reducing the amount of nitric oxide products. The statins also have beneficial effects on the neurotransmission of glutamate and have antioxidant and anti-excitotoxicity properties [6]. There have been two previous studies that showed the benefits of statins on ASD. The study was a double-blind, placebo-controlled RCT that examined the effectiveness of using statins as adjunctive therapy to risperidone in treating irritability symptoms in children with autism disorder. The trial involved 66 participants who were randomly assigned to either receive statins or a placebo in addition to risperidone. The study's findings showed that the use of statins as an adjunctive therapy led to a decrease in irritability symptoms in children with autism disorder [7]. Another study suggested that simvastatin may help reduce intra-myelin edema and improve cellular packing in patients with ASD and neurofibromatosis 1 [8].

Sphingosine 1-phosphate (S1P) acts as a lipid signaling molecule and it is related with Apo E in high density lipoprotein (HDL) complexes in central nervous system. S1P and its receptors have been reported to be useful targets in terms of pharmacological intervention in neurological area. FTY720/FingolimodTM, a sphingosine analogue, has been approved for the treatment of patients with relapsing multiple sclerosis. The action of mechanism of sphingosine analogue FTY720 was considered to suppress and block neuro-inflammation which are existed on astrocytes. Thus, S1P can be considered as a potential candidate for the treatment of various neuropsychiatric diseases [9].

In present research, it is proposed to investigate effects of atorvastatin on rodent model of ASD model via increasing S1P and anti-inflammatory actions.

## Materials and methods

Animals

Thirty male Wistar albino rats weighing 150-200 g and aged 10-12 weeks were used in this study. The experiments in this study were carried out in accordance with the National Institutes of Health's Guide for the Care and Use of Laboratory Animals (U.S.A). Animal Ethics Committee of Science University approved the present research (Demiroğlu Bilim University, Ethical number: 26211013).

Experimental procedures

Twenty-five mg/kg/day/rat of propionic acid (PPA) was administered intraperitoneally to 20 male Wistar rats, and 10 male Wistar rats were fed orally. Study groups were designed as follows: Group 1: Control Group (orally fed control, n=10); Group 2 (PPA+saline, n=10); Group 3 (PPA+Atorvastatin, n=10). All treatments were given for 15 days. The behavioral tests were started on 20 January 2022 and completed at the end of the 15-day period. All behavioral experiments were carried out between 10:00 and 15:00 hrs. Following the behavioral test, the animals underwent MR spectroscopy under IV ketamine anesthesia (50 mg/kg).

Behavioral tests

Three-chamber Sociability Test

**Figure 1:**
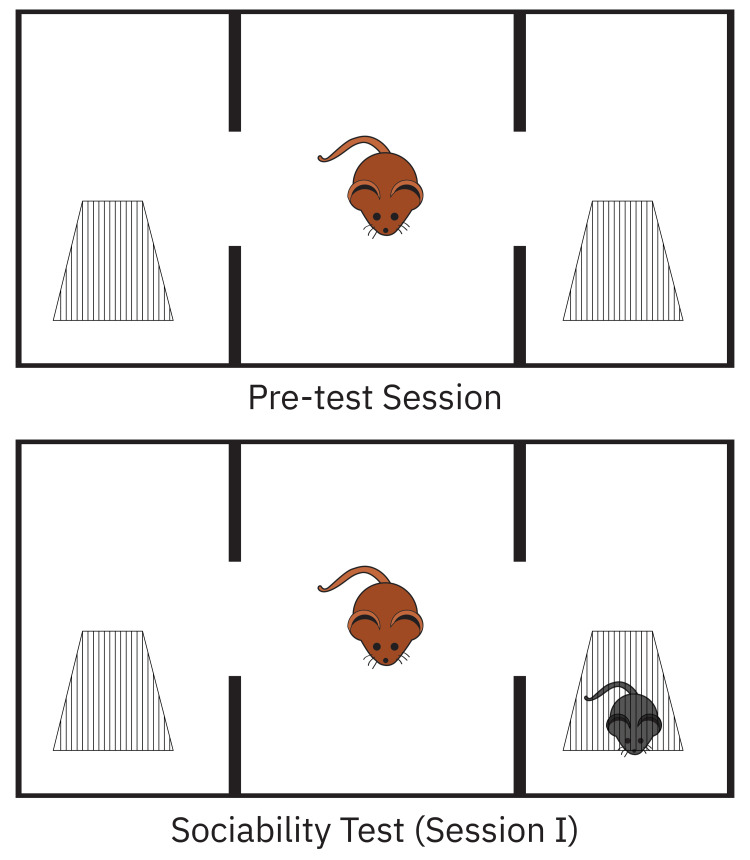
Demonstration of behavioral tests.

The sociability test was conducted with minor modifications as previously described elsewhere. In brief, a Plexiglas cage measuring 40 cm x 90 cm x 40 cm was divided into three equal regions measuring 40 cm x 30 cm x 40 cm each (Figure 1).

Open-field Test

To conduct the open field test, an open-aired box measuring 50 cm x 50 cm x 40 cm was utilized. At the start of the test, rats were gently placed in the center of the box and given the freedom to explore the arena for 5 minutes.

Passive avoidance learning (PAL)

During the PAL task, the rat is initially placed in the lighted chamber and allowed to explore the environment. After a predetermined amount of time, the door to the dark chamber is opened. If the rat enters the dark chamber, it receives an electric shock, which serves as a negative reinforcement.

MRI protocol, MR spectroscopy

Conventional MRI

All rats in the study were subjected to an examination using a 3.0-T MRI/MRS scanner manufactured by Siemens Healthcare. The examination utilized various conventional MR sequences including sagittal fast spin-echo T1 weighted imaging for location and axial spin echo T1 weighted imaging with a repetition time (TR) of 400ms and echo time (TE) of 11ms. Other parameters included a field of view (FOV) of 60mm, a matrix of 256 x 256, 2 excitation pulses, a bandwidth of 12.5kHz, a slice thickness of 1mm, an interslice gap of 0.2mm, and a total number of scan slices of 16.

MR spectroscopy

To perform 1H-MRS on the rats, an automated multivoxel 2D chemical shift imaging sequence was used, with a TR of 1000ms and a TE of 35ms. The phase encoding was set to x=24 and y=24, and the number of excitation pulses was one. The FOV had a diameter of 60mm, the slice thickness was 4mm, and the MRS voxel size was 1.87 x 1.87 x 4 mm^3^. The volume of interest was located within the appropriate striatum. The total time required to acquire the 2D 1H-MR spectrum was 580 seconds. The raw data obtained were processed using Siemens Healthcare's Magnetom software.

Hippocampus and cerebellum histopathology

Following washing in PBS, all sections were photographed using an Olympus C5050 digital camera. To calculate the GFAP immunostaining index, GFAP-positive cells were counted in three to four randomized sections of each rat at 40x magnification. The histopathological examinations were performed by the same investigator, who was blind to the study groups. The procedure was carried out in four sections per studied group, and an image analysis system (Image-Pro Express 1.4.5, Media Cybernetics, Inc. USA) was used for analysis.

Brain biochemical analysis

Commercially available rat enzyme-linked immunosorbent assay (ELISA) kits were utilized to measure the brain levels of various compounds, including TNF-α, Nerve Growth Factor (NGF), IL-17, IL-2, nuclear factor erythroid 2-related factor (NF-KB), NRF2, sphingosine 1 phosphate, and lactate.

Measurement of brain lipid peroxidation (MDA)

Malondialdehyde (MDA) levels as thiobarbituric acid reactive substances were used to determine lipid peroxidation in brain tissue samples (TBARS).

Measurement of brain protein levels

The Bradford method was used to determine the total protein concentration in brain samples, with bovine serum albumin serving as the standard.

Statistical Analysis

The statistical analyses were performed using SPSS version 23.0 (SPSS Inc., Chicago, IL, US). Normal distribution assumptions were checked using the Shapiro-Wilks normality test. Mann-Whitney U test and t-test were used for the comparison of patient and control groups for two independent samples based on distribution. ANOVA test and post hoc Tukey test were used to compare the numerical data of more than two groups. While the numeric data did not have a normal distribution, the Kruskal-Wallis test was used and a post hoc assessment was made by the Mann-Whitney test. The results are shown as the mean ±standard deviation. The value of p<0.05 was considered statistically significant.

## Results

Biochemical results

The levels of the brain MDA, TNF-α, IL-2, IL-17, and NF-KB were significantly higher in the PPA+saline group compared to the PPA+ atorvastatin group and control group. They were also significantly higher in the atorvastatin group compared to the control group. The levels of brain NGF, NRF2, and S1P1 were significantly higher in the control and atorvastatin groups compared to the saline group. The levels of NRF2 and S1P1 were found to be higher in the control group compared to the atorvastatin group. The lactate level was found to be higher in the saline group compared to the control and atorvastatin groups (Table 1).

**Table 1:** Comparisons of the results of the brain biochemical analysis between groups *ANOVA, **Kruskal Wallis a Control&Saline; p=0.00008, Atorvastatin&Saline; p=0.0003, Control&Atorvastatin; p=0.02 b Control&Saline; p=0.00006, Atorvastatin&Saline; p=0.01, Control&Atorvastatin; p=0.001 c  Control&Saline; p=0.001, Atorvastatin&Saline; p=0.02 Control&Atorvastatin; p=0.02 d Control&Saline; p=0.00008, Atorvastatin&Saline; p=0.0003, Control&Atorvastatin; p=0.02 e Control&Saline; p=0.0009, Atorvastatin&Saline; p=0.01, Control&Atorvastatin; p=0.001 f Control&Saline; p=0.02 Atorvastatin&Saline; p=0.04 g Control&Saline; p=0.009 Atorvastatin&Saline; p=0.004 h Control&Saline; p=0.0002, Atorvastatin&Saline; p=0.005, Control&Atorvastatin; p=0.003 i Control&Saline; p=0.0007, Atorvastatin&Saline; p=0.006, Control&Atorvastatin; p=0.04

	Control	PPA+saline	PPA+ Atorvastatin	Statistics
Brain MDA level (nmol/gr protein)	52.5 ± 1.7	177.04 ± 11.1	82.8 ± 6.5	F=8.66, p<0.0001*^a^
Brain TNF-alfa level (pg/mg protein)	13.1 ± 1.5	214.8 ± 15.04	108.6 ± 10.5	p<0.0001**^b^
Brain IL-2 level (pg/mg protein)	2.3 ± 0.08	256.7 ± 13.6	69.4 ± 5.3	p<0.0001**^c^
Brain IL-17 level (pg/mg protein)	221.7 ± 11.9	565.2 ± 23.8	348.5 ± 19.1	F=13.20, p<0.0001*^d^
Brain NF-KB level (pg/mg protein)	18.1 ± 1.05	194.2 ± 10.9	85.4 ± 7.7	p<0.0001*^e^
Brain Lactate level (mmol/100 g wet weight)	1.17 ± 0.02	3.24 ± 0.2	1.75 ± 0.3	p<0.05*^f^
Brain NGF level (pg/mg protein)	86.7 ± 4.9	44.1 ± 3.5	71.9 ± 2.2	F=7.54, p<0.001*^g^
Brain NRF2 level (pg/mg protein)	103.5 ± 9.8	51.2 ± 7.1	72.3 ± 3.05	F=9.33, p<0.001*^h^
Brain sphingosine 1 phosphate level (pg/mg protein)	121.04 ± 14.5	59.7 ± 5.2	98.2 ± 11.09	F=6.97, p<0.001*^i^

Behavioral tests

The scores of the Sociability test, open field test, and PAL were significantly lower in the saline group compared to the control and atorvastatin groups (Table 2).

**Table 2:** Comparison of the results of the behavioral tests between groups *ANOVA, **Kruskal Wallis a Control&Saline; p=0.0007 Atorvastatin&Saline; p=0.002 b Control&Saline; p=0.007 Atorvastatin&Saline; p=0.03 c Control&Saline; p=0.0005, Atorvastatin&Saline; p=0.008

	Control	PPA+saline	PPA+ Atorvastatin	
Sociability test: The spend of time with stranger rat percent (%)	62.8 ± 0.9	24.5 ± 1.4	50.6 ± 2.1	F=4.98, p<0.001*^a^
Open Field Test: Number of ambulation	13.1 ± 0.9	5.7 ± 1.04	10.3 ± 1.5	p<0.01*^b^
Passive avoidance learning (PAL) Latency (Sec.)	253.1 ± 28.2	115.3 ± 39.7	226.7 ± 13.8	F=10.11, p<0.0001*^a^

Hippocampus and cerebellum histopathology

**Figure 2:**
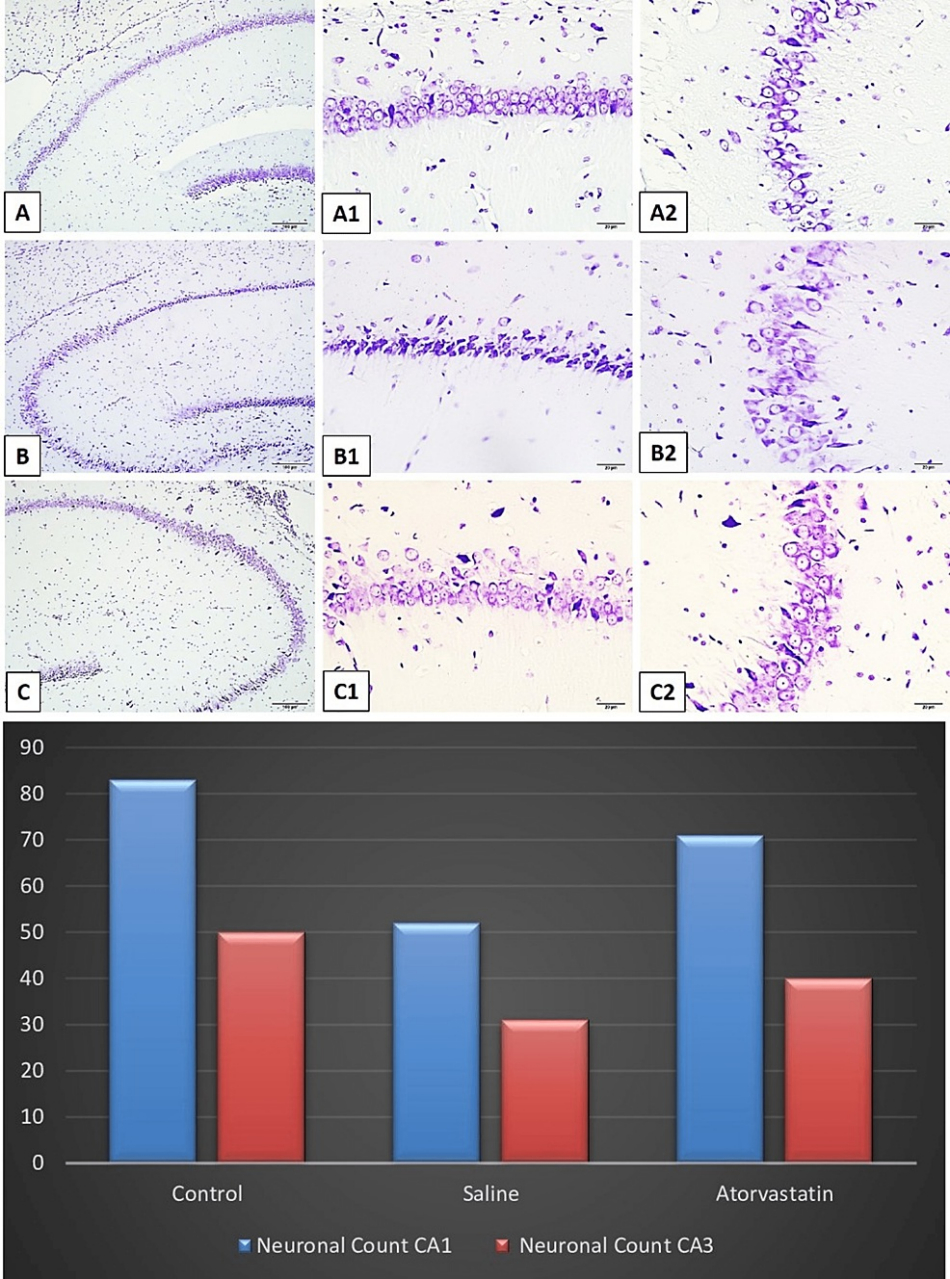
Light micrographs of cresyl violet-stained neurons in tissue sections of rat brain Figures A1-A2-A3 demonstrate normal pyramidal neuron counts in the CA1 and CA3 regions of the hippocampus. Figures B1-B2-B3 show the decreased amounts of neuron counts and dysmorphological changes in CA1 and CA3 regions. Figures C1-C2-C3 demonstrate the increased neural count and improved neural morphology changes in the CA1 and CA3 regions. The bars show the comparison of neuronal change counts between control, saline, and atorvastatin groups. The ANOVA test revealed that there are significant differences in the neuronal counts of CA1 ( F=15.66, p<0.0001).  Post hoc Tukey test showed that the control group and atorvastatin group had significantly higher neuronal counts of CA1 compared to the saline group (p=0.0001 and p=0.0009). The neuronal count of CA3 also was found to be higher in the control and atorvastatin groups compared to the saline group (F=11.12, p<0.01) (Post-hoc Tukey test: p=0.008 and p=0.03).

**Figure 3:**
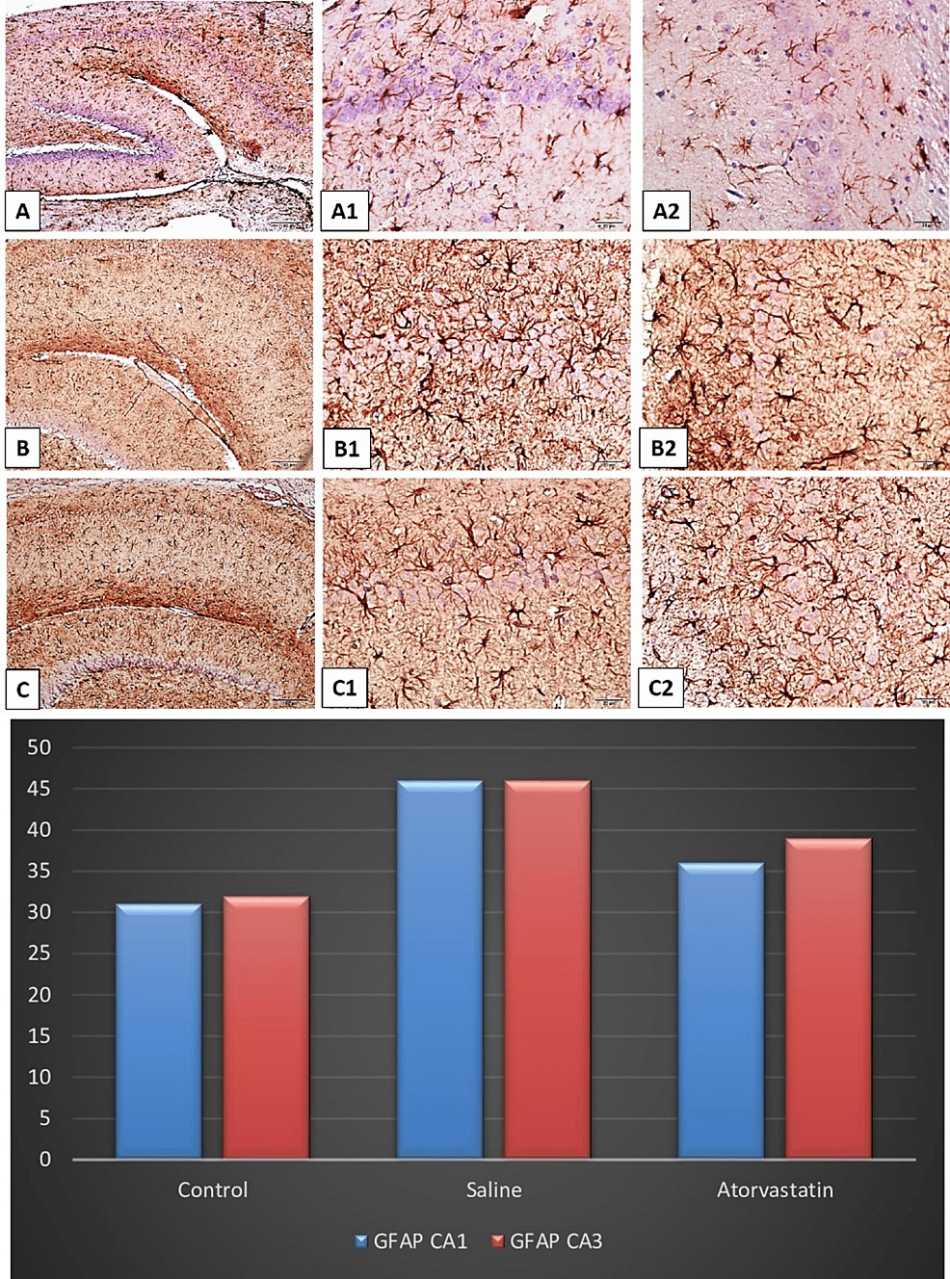
Light micrographs of cresyl violet-stained neurons in tissue sections of rat brain. The ANOVA test shows that there are significant differences in the neuronal counts of CA1 (F=11.38, p<0.01).  Post-hoc Tukey test showed that the control group and atorvastatin group had significantly higher neuronal counts of CA1 compared to the saline group (p=0.007 and p=0.04). The glial activity of CA3 was found to be significantly different between groups (F=9.76, p<0.01). The control group and atorvastatin group had a lower glial activity of CA3 compared to the saline group (p=0.004 and p=0.01). The control group also had a lower glial activity of CA3 compared to the atorvastatin group (p=0.04).

**Figure 4:**
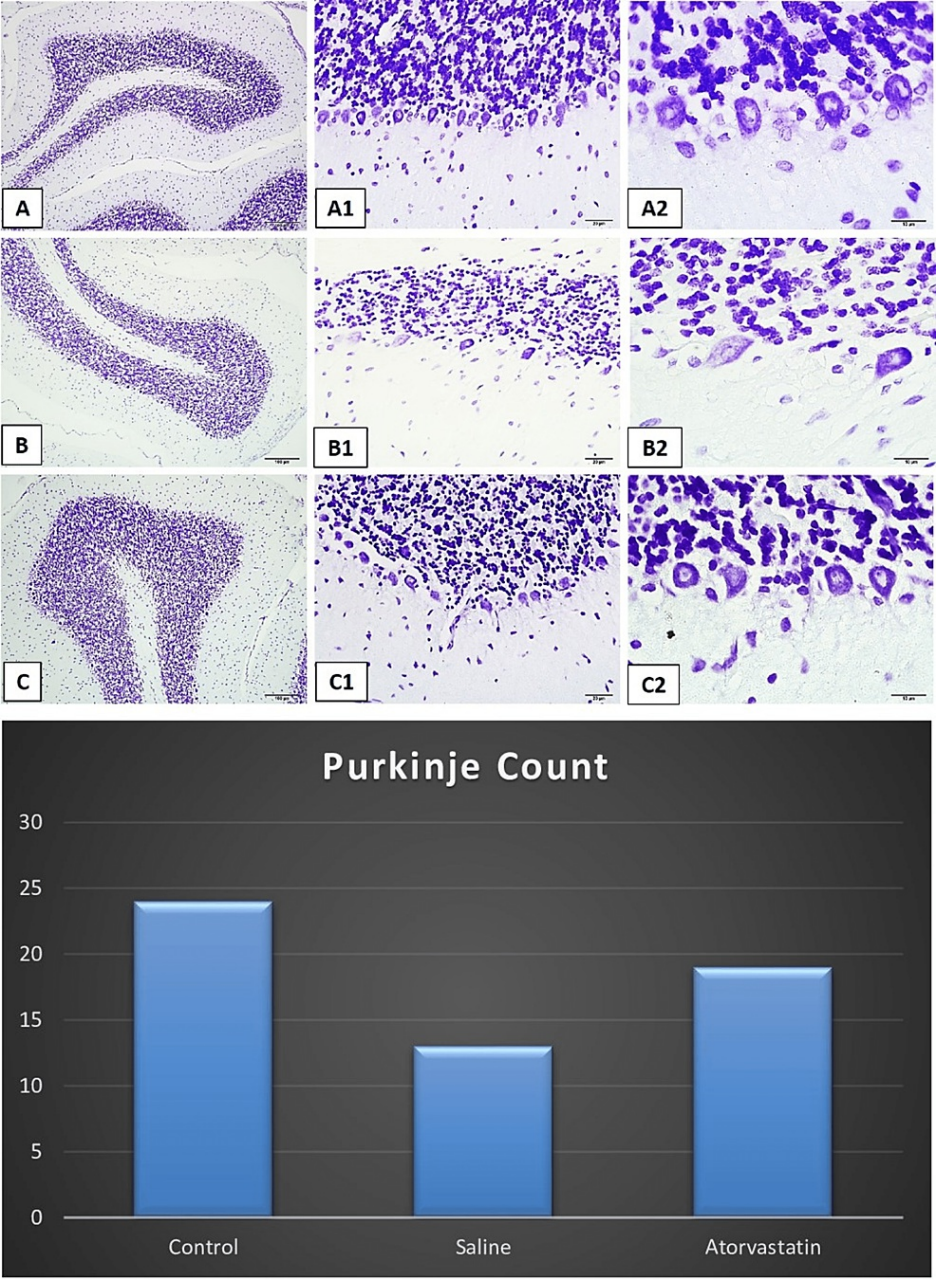
Light micrographs of cresyl violet-stained neurons in tissue sections of rat cerebellum Figures A1-A2-A3 demonstrate normal Purkinje neuron counts in the cerebellum. Figures B1-B2-B3 show the decreased amounts of Purkinje neurons and dysmorphological changes. Figures C1-C2-C3 demonstrate increased Purkinje neuron count in the cerebellum. The bars show the comparison of Purkinje neuron counts between the control, saline, and atorvastatin groups. The ANOVA test revealed that there were significant differences in the Purkinje neuron counts among groups ( F=7.87, p=0.003).  Post-hoc Tukey test showed that the control group and atorvastatin group had a significantly higher Purkinje neuron count compared to the saline group (p=0.01 and p=0.007).

**Figure 5:**
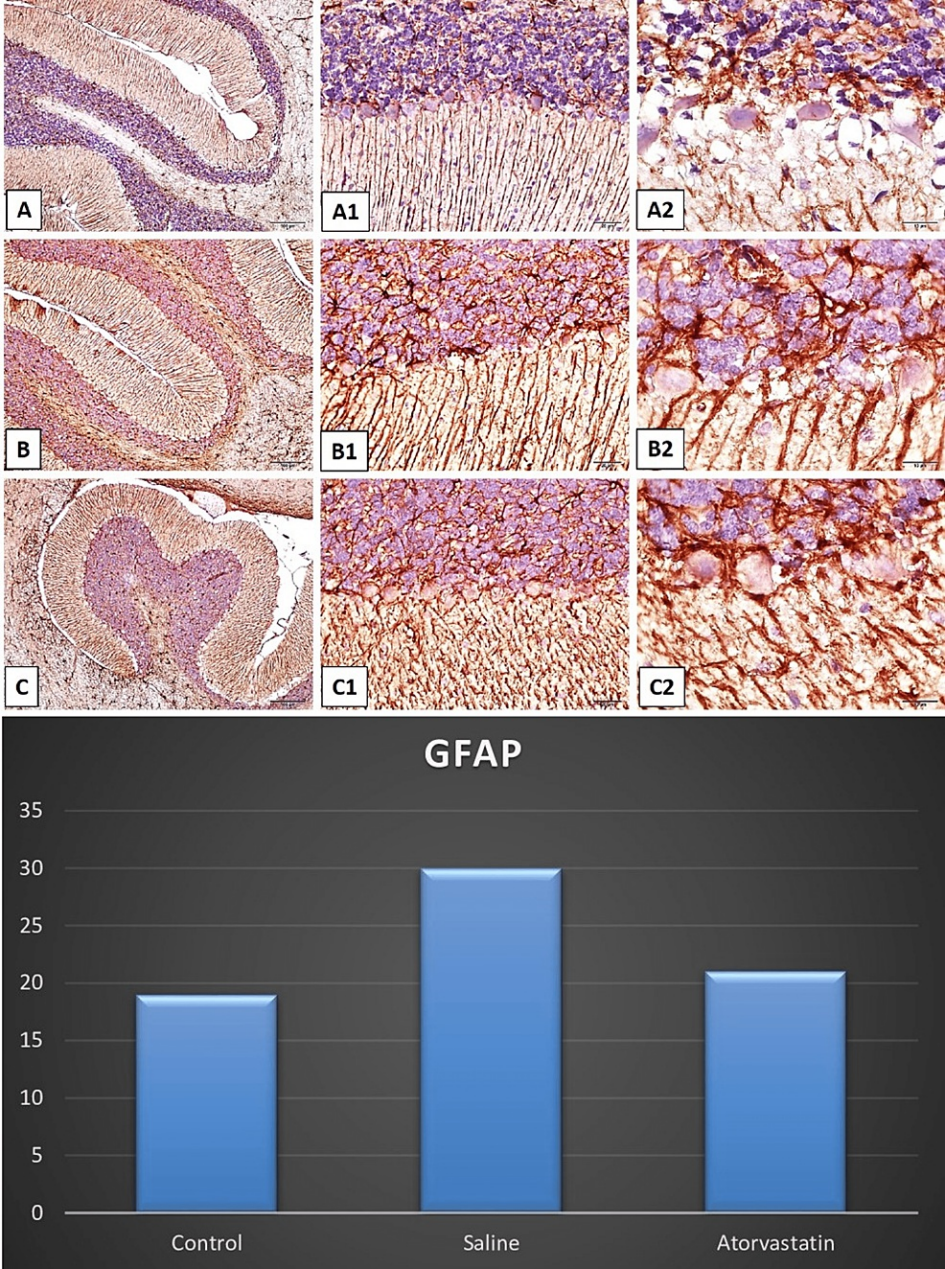
Light micrographs of cresyl violet-stained neurons in tissue sections of rat cerebellum Figures A1-A2-A3 demonstrate normal astrogliosis which was characterized by GFAP immunostaining (Brown staining) in the cerebellum. Figures B1-B2-B3 show the increased glial activity. Figures C1-C2-C3 demonstrate decreased glial activity in the CA1 and CA3 regions. The bars show the comparison of glial activity between control, saline, and atorvastatin groups. The ANOVA test showed that there are significant differences in neuronal counts of CA1 among groups ( F=5.92, p<0.01).  Post-hoc Tukey test showed that the control group and atorvastatin group had a significantly higher neuronal count of CA1 compared to the saline group (p=0.009 and p=0.047).

Neuronal counts CA1 and CA3 were higher in PA atorvastatin and control groups compared to the saline group. The control group and atorvastatin group had significantly higher neuronal count CA1 compared to the saline group. The control and atorvastatin groups had lower glial activity of CA3 compared to the saline group The control group also had a lower glial activity of CA3 compared to the atorvastatin group. The control group and atorvastatin group had significantly higher Purkinje neuron counts compared to the saline group. The control group and atorvastatin group had significantly higher neuronal count CA1 in the cerebellum compared to the saline group. Figures 2-5 demonstrate the histopathology examinations (Figure 2-5).

MR spectroscopy

**Figure 6:**
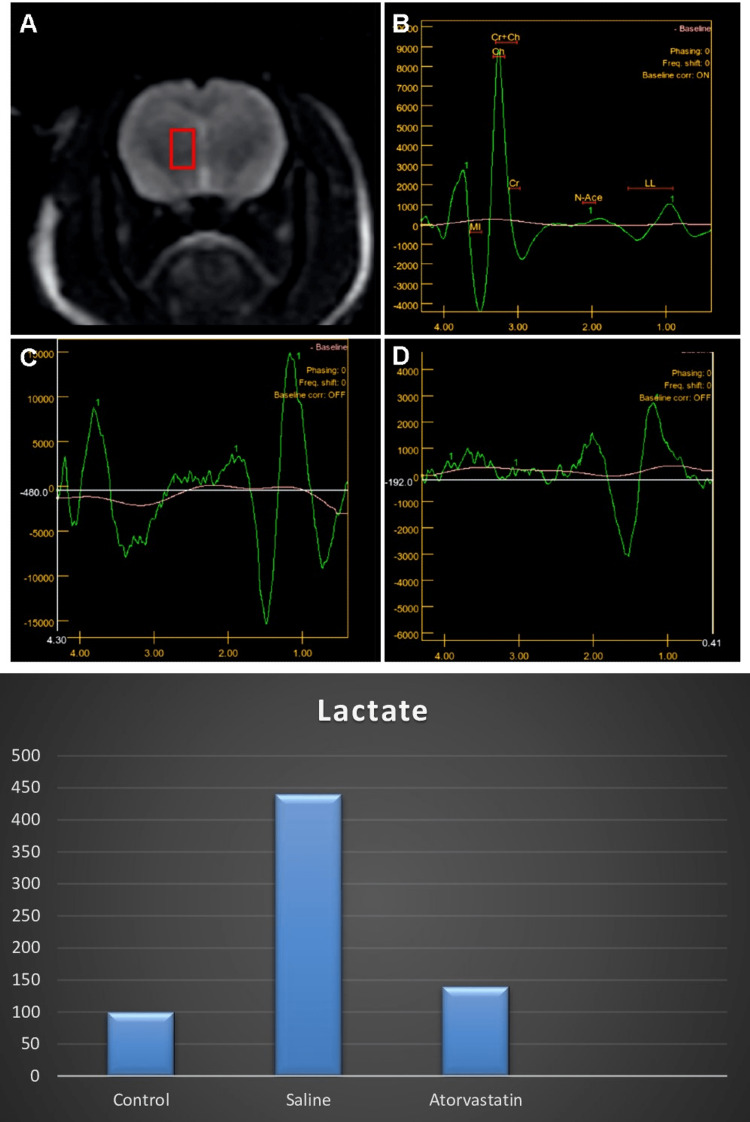
MR spectroscopy assessment of rat brain The red box demonstrates the chosen area. Figure B demonstrates lactate levels of control rats. Figure C shows the increased lactate levels in the saline group. Figure D shows the decrease of lactate levels in the atorvastatin group. The bars show the comparison in lactate levels between the control, saline, and atorvastatin groups. The ANOVA test showed that there were significant differences in lactate levels among groups (F=15.32, p<0.0001). Post-hoc Tukey test showed that the control group and atorvastatin group had significantly higher lactate levels compared to the saline group (p=0.0001 and p=0.0007). The control group also had higher lactate levels compared to the atorvastatin group (p=0.0004).

The control group and atorvastatin group had significantly lower lactate levels compared with the saline group. The control group also had lower lactate levels compared with the atorvastatin group (Figure 6).

## Discussion

In the present research, it was demonstrated that atorvastatin had beneficial effects on behavioral tests and improved outcomes on neurobiological, histopathological, and imaging results compared to the saline group in the PPA-induced rat model of ASD. Further clinical research is needed to support our preliminary results.

PPA can pass the blood-brain barrier and affect several systems in the central nervous system. Furthermore, high amounts of PPA (e.g. 4 μl of 0.26 M solution) are considered to have toxic properties such as seizures, some types of movement disorders, and as well as developmental delays [10]. The intracerebroventricular administration of PPA into rat brains was demonstrated to increase the levels of IL-6, TNF-α, and interferon-γ cytokine levels [11]. In the present research, we also succeeded to demonstrate higher brain levels of MDA, TNF-α, IL-2, and IL-17 in the saline group compared to the control and atorvastatin groups. Atorvastatin was shown to have anti-inflammatory and immunomodulatory effects on several tissues besides its lipid-lowering effects [12]. Recently, it was shown that statins could modulate the suppressive functions and the recruitment of T-reg cells in several tissues including the brain [13]. Thus, these results supported the findings that atorvastatin has benefits on the neurobiology of ASD via anti-inflammatory effects and decreasing oxidative stress. 

NGF was reported to have a key role in neuron survival. The literature on the association between NGF and ASD is limited. Lu and coworkers reported nonverbal deficit could be associated with NGF single-nucleotide [14]. NRF2 and its associated pathways were known as the “guardian of redox homeostasis.”[15]. The activation of the NRF2-ARE pathway is an important component of the upregulation of many downstream products which are particularly associated with oxidative stress [16]. Thus, it can be clearly said that NRF2 is an important factor against to oxidative system in brain tissue. In the present study, both NGF and NRF2 were found to be lower in the PPA+saline group, and the atorvastatin group was found to have significantly higher levels of NGF and Nrf2 compared to the PPA+saline group. Our results indicate that atorvastatin ameliorated both inflammation and oxidation in brain tissue.

S1P1 is a form of the membrane lipid sphingosine and plays a critical role for G-protein coupled receptors. S1P1 is considered to have signaling properties as both autocrine and paracrine fashions; thus it can be said S1P1 can affect both feeding back on the cell of origin or other cell types [17]. S1P1 was also reported to have particular roles in the neurons and as well as vascular systems’ structure during embryogenesis. It also has essential roles for various neurotransmitter systems such as glutamatergic and acetylcholinergic systems [18,19]. FTY720/FingolimodTM, an immunosuppressive sphingosine analogue, is the first-line treatment for relapsing multiple sclerosis [20]. Recently, De Simone and his coworkers demonstrated the beneficial effects of Fingolimod (FTY720) - a non-selective Sphingosine 1-Phosphate Receptor ligand) on the social behaviors of mice in which an experimental autism model was created [21]. In their experimental study, Igarashi and colleagues demonstrated that statins activate S1P1 receptors and enhance endothelial cell responses to HDL-associated sphingolipids [22]. Regarding the effects of atorvastatin on S1P1 in our study and as well as previous literature mentioned above, atorvastatin can be considered as a good candidate for protecting the neuro-inflammation and mitochondrial dysfunction in ASD.

The hippocampus plays a critical role in the etiology of ASD. The hippocampus volume was reported to be decreased in patients who suffered from ASD in childhood episodes. The volume of the hippocampus was reported to be decreased bilaterally among adult patients with ASD [23]. Chaddad and colleagues found that using a novel MRI multi-scale image texture analysis to quantify the spatial heterogeneity of brain tissues resulted in significant changes in hippocampal volume [24]. Concurring evidence shows that ASD may be significantly associated with a disorder in glial cells. GFAP was reported to be more in the brain tissue of ASD compared to healthy controls. Moreover, reduced microbiota could be associated with impaired microglial proliferation [25]. The results of the present study showed that atorvastatin might improve cell count and microglial formation in a PPA-induced autism model. We argue that atorvastatin may lead to the prevention of neuro-inflammation and may provide significant effects on the core symptoms of ASD.

Cerebellar dysfunctions were reported to have a role in the etiology of ASD. Although the exact pathophysiology is unknown, cerebellar abnormalities were reported to be associated with core symptoms of ASD. Moreover, there have been reports that showed decreased cerebellar volume in patients with ASD compared to healthy controls [26]. Purkinje cells are also considered to be an interesting factor in ASD etiology. The decrease in Purkinje cells in ASD subjects was reported to be associated with astroglial activation (GFAP) and decreased GABAergic signaling pathway and increased glutamatergic activity [27]. The results of the present research supported the literature and also showed that atorvastatin might have protective effects on neurons subjected to PPA-induced damage in rat cerebellum.

Mitochondrial disease is considered to have a place in the etiology of ASD [28]. Mitochondrial dysfunctions may have a role in the development of core cognitive and behavioral symptoms. In vivo studies showed that increased brain lactate levels indicated mitochondrial disease in ASD with magnetic resonance spectroscopy (1H MRS) [29]. A current study demonstrated that lactate levels were increased in ASD patients, thus supporting our findings of mitochondrial dysfunction in ASD [30]. The present study showed increased lactate levels in the saline group by both tissue investigation and magnetic resonance spectroscopy imaging. Furthermore, atorvastatin has a beneficial effect on decreasing lactate levels compared to the saline group. Thus, we argue that atorvastatin can have a favorable effect on possible mitochondrial dysfunction in ASD brains.

## Conclusions

The present study is the first to demonstrate the beneficial effects of atorvastatin on the rat model of ASD. Atorvastatin seems to have favorable effects on brain tissue which were provided with biochemical, histopathological, and imaging assessments in the present study. Current findings suggest that atorvastatin increases sphingosine-1-phosphate levels and decreases inflammatory actions which characterize the autism rodent model implemented in this study. These preliminary results have to be confirmed by further experimental and clinical studies.
